# Brain involvement in Alström syndrome

**DOI:** 10.1186/1750-1172-8-24

**Published:** 2013-02-13

**Authors:** Valentina Citton, Angela Favaro, Vera Bettini, Joseph Gabrieli, Gabriella Milan, Nella Augusta Greggio, Jan D Marshall, Jürgen K Naggert, Renzo Manara, Pietro Maffei

**Affiliations:** 1 Neuroradiology Unit, IRCCS San Camillo Hospital Venezia, Padova, Italy; 2 Department of Neurosciences, University of Padua, Padova, Italy; 3 Internal Medicine 3, Department of Medicine, University Hospital of Padua, Padua, Italy; 4 Neuroadiology Unit, University Hospital of Padua, Padua, Italy; 5 Pediatric Endocrinology Unit; Department of Pediatrics, University Hospital of Padua, Padua, Italy; 6 The Jackson Laboratory, Bar Harbor, Maine, USA; 7 Neuroradiology Unit, University of Salerno, Ospedale San Camillo, Venice, Italy

**Keywords:** Alström syndrome, MRI, DTI, VBM

## Abstract

**Background:**

Alström Syndrome (AS) is a rare ciliopathy characterized by cone–rod retinal dystrophy, sensorineural hearing loss, obesity, type 2 diabetes mellitus and cardiomyopathy. Most patients do not present with neurological issues and demonstrate normal intelligence, although delayed psychomotor development and psychiatric disorders have been reported. To date, brain Magnetic Resonance Imaging (MRI) abnormalities in AS have not been explored.

**Methods:**

We investigated structural brain changes in 12 genetically proven AS patients (mean-age 22 years; range: 6–45, 6 females) and 19 matched healthy and positive controls (mean-age 23 years; range: 6–43; 12 females) using conventional MRI, Voxel-Based Morphometry (VBM) and Diffusion Tensor Imaging (DTI).

**Results:**

6/12 AS patients presented with brain abnormalities such as ventricular enlargement (4/12), periventricular white matter abnormalities (3/12) and lacune-like lesions (1/12); all patients older than 30 years had vascular-like lesions. VBM detected grey and white matter volume reduction in AS patients, especially in the posterior regions. DTI revealed significant fractional anisotropy decrease and radial diffusivity increase in the supratentorial white matter, also diffusely involving those regions that appeared normal on conventional imaging. On the contrary, axial and mean diffusivity did not differ from controls except in the fornix.

**Conclusions:**

Brain involvement in Alström syndrome is not uncommon. Early vascular-like lesions, gray and white matter atrophy, mostly involving the posterior regions, and diffuse supratentorial white matter derangement suggest a role of cilia in endothelial cell and oligodendrocyte function.

## Background

Alström syndrome (AS; OMIM#203800) is an autosomal recessive rare disorder caused by mutations in *ALMS1* (chromosome 2p13), with a frequency estimated at <1:100.000 [1]. The syndrome is multisystemic and major phenotypes include cone-rod retinal dystrophy leading to blindness, hearing loss, obesity, type 2 diabetes mellitus (T2DM), dilated cardiomyopathy, and progressive hepatic and renal failure [1]. Fibrosis in multiple organs has been described [1]. There is considerable variation in the phenotypic spectrum both among and within different families [1,2].

ALMS1 is ubiquitously expressed, but the function remains unknown. At least one isoform of ALMS1 localizes to the centrosomes and basal bodies of ciliated cells. Studies in murine and cellular models suggest that defects in ciliogenesis and/or structure, function, or maintenance of cilia underlie the pathogenesis of AS [2,3].

ALMS1 is widely expressed in most regions of the brain (http://www.brain-map.org/). Most AS patients demonstrate normal intelligence, although some reports indicate delayed early psychomotor and intellectual development. Hearing and vision deficits probably contribute to learning delays seen in some young children with AS. Motor milestones are typically delayed by 1–2 years and there may be deficits in coordination, balance, and fine motor skills. A range of autism-spectrum behavior and clonic-tonic or absence-seizure activity has been observed in some cases. Major depression, hyperphagia, obsessive-compulsive, and psychotic behaviors have been noted, particularly in adults [1,2]. The mechanism underlying the neurological involvement remains unknown in AS and brain imaging has not been studied.

The aim of this study is to specifically investigate central brain involvement by conventional Magnetic Resonance Imaging (MRI), Voxel Based Morphometry (VBM) and diffusion tensor imaging (DTI) in a consistent group of pediatric and adult patients affected by AS.

## Patients and methods

### Subjects

Twelve patients (mean-age 22 years; range: 6–45, 6 females) affected with Alström Syndrome were recruited from our Department of Internal Medicine (11 patients) and from The Jackson Laboratory (1 patient). All the patients met the diagnostic criteria for AS based on genetic analysis and clinical observations [1] and had a normal intellectual quotient.

Nineteen age- and gender-matched unrelated healthy subjects or patients referred to neuroimaging for headache with no history of prematurity, head trauma, neurological or psychiatric disease and neurosurgery represented our control group (mean age 23 years; range: 6–43; 12 females).

Two AS patients and 1 control were left-handed according to the Edinburgh handedness inventory [4]. The exclusion criteria were the presence of contraindications to MRI and age < 6 years in order to avoid sedation. The study was approved by the University Hospital of Padua Ethics Committee and the Institutional Review Board of The Jackson Laboratory. Written informed consent was obtained from patients or their parents.

All subjects underwent a complete physical and neurological examination including neurosensory analysis (retinopathy and hearing loss) and were investigated for cardiovascular risk factors and comorbidities (obesity, T2DM, cardiomyopathy, and hyperlipidemia). Clinical findings are summarized in Table 1.

**Table 1:** **Epidemiological**, **genetic and clinical findings of AS patients in the present study**

**ID**	**Age(y),Sex**	**Genotype(exon)**	**Vision deficit**^**1**^	**Hearing deficit**^**2**^	**Overweight**^**3**^	**Hypertension**^**4**^	**Heart Ejection fraction**^**5**^	**Dyslipidemia**	**DM2**^**6**^	**Hepatosteatosis**	**Psisichiatric disorders P**
1	6, M	heterozygosis 8 and 16	+	-	++	-	normal	-	+	-	-
2	9, M	heterozygosis 8 and 16	+	+	++	-	normal	-	+	-	-
3	11, F	heterozygosis 8	+	+	+	-	normal	-	+	+	-
4	12, F	heterozygosis 8	+	+	+	-	normal	-	+	-	-
5	13, F	heterozygosis 8 and 16	++	+	+	+	normal	+	++	+	-
6	18, M	heterozygosis 8	+	+	-	-	normal	-	+	+	-
7	23, M	heterozygosis 10	+	+	+	+	25% *	+	++	+	-
8	26, F	heterozygosis 16	++	+	++	+	47%	-	++	+	depression
9	29, F	heterozygosis 8 and 16	++	+	+	-	normal	+	+	+	depression
10	31, M	homozygosis 8	++	+	+	+	normal	+	-	+	anxiety,depression bulimia^7^
11	43, M	homozygosis 8	++	+	+	+	normal	+	-	+	paranoid personality, disorder ^8^
12	45, F	homozygosis 10	++	+	-	+	normal	+	++	+	-

^1^Vision Deficit: + = severe ipovision (< 1/20); ++ = blindness.

^2^Hearing Deficit: - = normal hearing; + = hearing deficit >20dB;

^3^Overweight: - = normal weight (Body Mass Index 18.5 to 25), + = overweight (Body Mass Index 25 to 30); ++ = obesity (Body Mass Index > 30).

4Hypertension: - = normotensive; + = under antihypertensive treatment.

5Heart ejection Fraction: normal value > 55%.

6++ = diabetes mellitus type 2, + = insulin resistance or hyperinsulinemia or impaired glucose tolerance.

7 Under therapy with valproic acid, bromazepam and Olanzapine.

8 Under therapy with valproic acid, quetiapine, risperidone, paroxetine.

* Dilated Cardiomyopathy.

In particular, one patient (# 7) presented with mild cardiac impairment since infancy.

All our subjects manifested a mild developmental delay.

### Image acquisition

For 11 patients and all controls, MRI scans were performed at 1.5T (Achieva, Philips Medical Systems, Best, the Netherlands) with a standard quadrature head coil.

The MRI study protocol included:

1. 3DT1 weighted imaging (repetition time/echo time: 20/3.8 msec; flip angle: 20°; slice thickness: 1 mm, pixel size: 0.66 × 0.66 mm);

2. Fluid-attenuated inversion-recovery (FLAIR, repetition time/echo time: 10000/140 msec; echo train length: 53; flip angle: 90°; slice thickness: 5 mm, pixel size: 0.89 × 0.89 mm);

3. Diffusion tensor images acquired with a single-shot echo planar diffusion weighted imaging according to the following parameters: repetition time/ echo time: 10389/80 ms, echo train length: 59; acquisition matrix: 128 × 128, reconstructed voxel: 1.75 × 1.75 × 2 mm, SENSE p reduction: 2, slice thickness: 2 mm without gap, Number of Excitations: 2.

The axial sections covered the whole brain including the cerebellum. The diffusion sensitizing gradients were applied along 32 non-collinear gradient encoding directions with maximum b = 800 s/mm2. One additional image without diffusion gradients (b = 0 s/mm2) was also acquired.

Conventional MR images of patient #5 were collected in another Center with a 1.5T scanner; this patient was excluded from DTI and VBM analysis.

### Image analysis

#### Grey matter preprocessing

We used the optimized VBM protocol available in the statistical parametric mapping software (SPM8, http://www.fil.ion.ucl.ac.uk/spm/) [5]. The protocol consisted of the following: a study specific grey matter template was built from the 30 segmented native images affine-registered to the ICBM-152 grey matter template. The native segmented images were then non-linearly normalized onto this template, introducing a 'modulation' for distortions due to the non-linear component of the transformation by dividing each voxel of each registered grey matter volume image by the Jacobian of the warp field. The modulated normalized grey matter volume images were then smoothed with an isotropic Gaussian kernel with a sigma of 3 mm.

For statistical analyses, we used parametric t-test as implemented by SPM8, using age as a covariate of no interest. Results for grey matter were considered significant for p < 0.06, after correction for multiple comparisons using an initial cluster-threshold at p < 0.001 uncorrected (minimum size = 20 voxels). Region of interest analyses for hippocampal regions was performed using WFU-PickAtlas 3.0.3while contrasts were analyzed using SPM [6].

#### White matter preprocessing

All DTI data were preprocessed by the Oxford Center for Functional MRI of the Brain (FMRIB)’s Diffusion Toolbox (FDT) within FMRIB’s Software Library (FSL; http://www.fmrib.ox.ac.uk/fsl/) [5]. First, the diffusion-weighted volumes were aligned to its corresponding non-diffusion-weighted (b0) image with an affine transformation to minimize image distortion from eddy currents and to reduce simple head motion. Then, non-brain tissue and background noise were removed from b0 image using the Brain Extraction Tool. After these steps, the diffusion tensor for each voxel was estimated by the multivariate linear fitting algorithm, and the tensor matrix was diagonalized to obtain its three pairs of eigenvalues (L1, L2, L3) and eigenvectors. Maps of fractional anisotropy (FA), mean diffusivity (MD), axial diffusivity (L1: first eigenvalue), radial diffusivity (RA: average of the second and third eigenvalue) were then generated.

Whole brain analysis of FA images was performed by using Tract-Based Spatial Statistics (TBSS). In brief, FA maps of all subjects were first realigned to a common target and then the aligned FA volumes were normalized to the Montreal Neurological Institute standard space (MNI152). Thereafter, the registered FA images were averaged to generate a cross-subject mean FA image, and then the mean FA image was applied to create a mean FA skeleton which represents the main fiber tracks and the center of all fiber tracts common to the group. The mean FA skeleton was further thresholded by a FA value of 0.2 to exclude peripheral tracts where there was significant inter-subject variability and/or partial volume effects with grey matter. Following the thresholding of the mean FA skeleton, the aligned FA data of each participant was projected onto the mean skeleton to create a skeletonized FA map, by searching the area around the skeleton in the direction perpendicular to each tract, and finding the highest local FA value, and then assigning this value to the corresponding skeletal structure.

To identify FA differences between AS patients and controls, the skeletonized FA data were fed into the voxel-wise statistics analysis which is based on non-parametric approach utilizing permutation test theory. The testing was performed by the FSL randomise program, which uses 5000 random permutations.

Age was entered into the analysis as a covariate. Threshold-Free Cluster Enhancement (TFCE) approach [7] was used to obtain the significant differences between two groups at p ≤ 0.05, after accounting for multiple comparisons by controlling for family-wise error (FWE) rate.

## Results

### Conventional MRI

None of the controls presented any parenchymal abnormality except for an 18-year-old control who had brain MRI because of headache and presented with mild ventriculomegaly and a small aspecific white matter T2 hyperintense lesion on the left frontal lobe.

**Figure 1:**
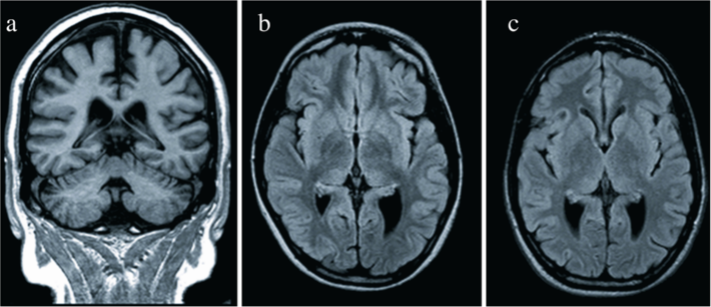
**Brain MRI: coronal T1**-**weighted (a) and axial FLAIR (b, c) images of Alström syndrome patients. a**) 31-year-old male: mild brain atrophy with enlargement of ventricles and subarachnoid spaces; **b**) 11-year-old girl: enlargement of the left atrium of lateral ventricle; **c**) 18 year-old boy: bilateral dilated atria of the lateral ventricle.

**Figure 2:**
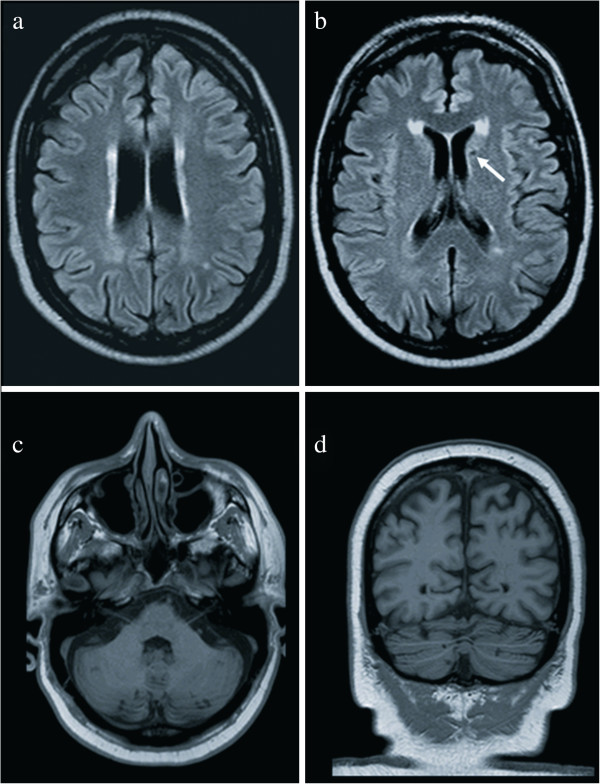
**Brain MRI of a 43-year-old male patient. **Axial FLAIR (**a**, **b**); axial (**c**) and coronal (**d**) T1 weighted images. **a**,**b**) hyperintense rim bordering the margin of both lateral ventricles and a small lacune in the left head of the caudate (arrow); **c**, **d**) multiple small hypointense lesions in the cerebellar cortex.

In the AS group, MRI revealed mild ventricular enlargement (two patients), mild ventricular enlargement with increased subarachnoid spaces (one patient), isolated enlargement of the left lateral ventricle (Figure 1). Ventricular enlargement mostly involved the atria and the posterior horns of the lateral ventricles; a mild increase of the tip of the temporal horn was observed in the 31-year-old male. Three AS patients (25%) presented with periventricular white matter hyperintensity; one patient also presented with several small cortical cerebellar lesions and a left caudate lacune (Figure 2).

### Voxel-based morphometry

**Figure 3:**
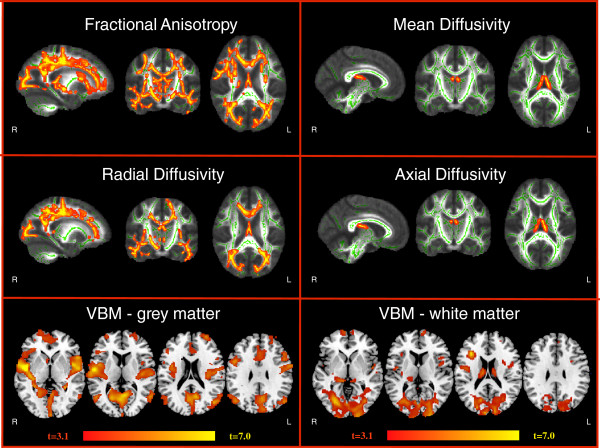
**TBSS and VBM analysis findings: **colored areas show significant differences of Fractional Anisotropy, Radial Diffusivity, Axial Diffusivity, Mean Diffusivity, gray matter and white matter volume between Alström patients and controls. Diffuse myelin supratentorial involvement and regions of symmetric parenchymal atrophy.

Significantly lower grey matter volume was found in AS patients compared to controls (0.878 ± 0.122 vs. 1.023 ± 0.117 liters; F(1,25) = 18.50; p < 0.001 using age, gender and global intracranial volume as covariates, Figure 3) as described in Table 2. Similarly, white matter volume decrease (0.591 ± 0.078 vs. 0.685 ± 0.108 liters; F(1,25) = 13.85; p = 0.001 using age, gender and total intracranial volume as covariates, Figure 3) was detected, especially in the posterior regions.

**Table 2:** Differences in regional gray and white matter volumes between AS patients and controls

**Brodmann**		**Anatomical labels**^**a**^	**No.of voxels**	***p*-Value(FWE corrected)**^**b**^	**MNI coordinates(mm)c**
Grey Matter					
BA22	LH	Sup Temporal	35728	0,0001	−46 0 -4
	LH	Cerebellum	793	0,0001	−16 -40 -56
BA39	RH	Middle temp gyrus	1187	0,0001	46 -74 22
BA10	RH	Sup front Gyrus	1089	0,0001	32 58 22
BA39	LH	Angular gyrus	860	0,0001	−46 -72 32
White Matter					
BA17	RH	Middle Occipital	6181	0,0001	−26 -98 6
BA19	LH	Middle Occipital			−24 -74 2
BA4	RH	Precentral	3200	0,0001	42 -22 62
BA4	LH	Middle cingulum			−8 -32 46
	RH	Cerebellum	900	0,0001	24 -40 -48

^a^Anatomical labels were obtained using AAL Toolbox; sup = superior; temp = temporal; front = frontal.

^b^ Statistical significance after correction for multiple comparisons (Family Wise Error voxel-level).

^c^ Montreal Neurological Institute coordinates of the voxel of maximal statistical significance within each region.

LH = left hemisphere; RH = right hemispher.

By means of ROI analysis for hippocampal regions, a significant differences in GM volume for both left and right hippocampus was found. The difference persisted after FDR correction only in the right hippocampus (p = 0.046). ROI analysis conducted using total GM as covariate showed clusters of significant difference in both left (cluster-level FWE-corrected, p = 0.033, peak: -12, -38, 10) and right hippocampus (cluster-level FWE-corrected, p = 0.050, peak: 12, -38, 10).

### DTI

TBSS analysis showed regions of significantly reduced fractional anisotropy and increased radial diffusivity in AS patients compared to controls (Figure 3). In particular, the optic and acoustic radiation, body of the corpus callosum, cingulum, fornix, centrum semiovale, limb of the internal and external capsule were bilaterally affected. Noteworthy, DTI white matter signal abnormalities also extended to those supratentorial regions that appeared normal on conventional MRI sequences. There were no white matter regions where controls had significantly lower fractional anisotropy values or increased radial diffusivity compared to AS patients.

Axial diffusivity and mean diffusivity were significantly increased in the fornix of AS patients compared with controls (Figure 3). There were no white matter regions where controls had significantly higher mean diffusivity and axial diffusivity.

## Discussion

This study showed that brain involvement is not uncommon in AS patients. On conventional MRI sequences 50% of AS patients presented brain abnormalities including subtle signs of atrophy, periventricular WM abnormalities or lacune-like lesions. Furthermore, VBM revealed white and grey matter volume decrease more evident in the posterior regions while diffusion tensor imaging showed diffuse supratentorial white matter abnormalities involving also the regions that appeared normal on conventional imaging.

So far, neuroimaging data in AS are very scarce. Brain involvement was reported in a 6-year-old girl with AS presenting with aphasia since birth and mild right hemiparesis noted three months before admission [8]. Her brain CT revealed atrophy of the left hemisphere, that according to the authors could have been a coincidental association. Among our patients we had no cases of hemiatrophy, although one patient presented with diffuse mild atrophy and three presented with uni- or bilateral ventricular enlargement without significant increase of subarachnoid spaces. In all cases the ventricular enlargement was more prominent in the posterior part of the lateral ventricles. Likewise, VBM showed a significant white matter and grey matter volume reduction in AS patients, more evident in the posterior cerebral regions.

Although posterior lateral ventricle enlargement has been recognized in several clinical conditions, none of the patients had history of prematurity, peripartum anoxic-ischemic events or head trauma. Interestingly, the posterior horns lie very close to the optic pathway which is primarily affected in AS. A selective atrophy of the optic pathway, therefore, could have contributed to the atrophy of the posterior part of the cerebral hemispheres, as observed in congenital and early blindness [9,10].

In Bardet Biedl Syndrome (BBS) patients, focal temporal lobe and midline orbitofrontal lobe grey matter volume loss and hippocampal dysplasia were related to a severe and global impact of ciliary dysfunction on forebrain development [11,12]. Our study disclosed a more diffuse lower grey matter volume without the specific distribution detected in BBS patients. Moreover, no AS patient met the neuroimaging criteria for hippocampal dysplasia. These findings seem to reveal that brain involvement in these two ciliopathies is dissimilar, thus providing some structural basis for the known different cognitive impairment.

Three AS patients presented with periventricular white matter hyperintensity. This type of lesion is frequently observed in normal subjects but at a much older age [13] and is attributed to altered hemodynamics since periventricular area is supplied by noncollateralizing ventriculofugal vessels arising from subependymal arteries. Since up to 60% of AS subjects might present with infant cardiomyopathy and sudden onset of cardiac heart failure in their first month of life [1], parenchymal brain changes might root from heart-related brain hypoxia.

However, cardiac function usually improves before the age of 3, while parenchymal changes seem to appear in AS patients after the age of 30, thus suggesting a different pathogenic mechanism. T2DM, hypercholesterolemia, obesity and heart failure, which are frequent comorbidities in AS, might all contribute to decreased cerebral blood flow, leading to early periventricular white matter hyperintensity in AS patients.

In addition to periventricular white matter lesions, the oldest male patient (43-year-old) had several lesions consistent with small infarcts, almost all in the cerebellar cortex. The pathogenesis of these lesions is unclear. Global hypoperfusion and cardioembolism preferentially involve the supratentorial structures and do not affect almost exclusively the cerebellum as in our patient [14]. On the other hand, bilateral vertebral embolic sources in the young (such as bilateral vertebral artery dissection) do not usually occur without symptoms and would not explain the lesion observed in the left internal carotid artery territory (caudate nucleus). Alternatively, the preferential involvement of the posterior circulation could be related to AS itself. Ciliary involvement in endothelial function has been demonstrated [15].

The presence of primary cilia in arteries with oscillatory flow suggests a region-specific distribution.

These aspects could underlie a region-specific increased susceptibility to risk factors present in AS. Indeed, other inherited conditions (e.g. Fabry disease) present with an increased risk of stroke in the posterior circulation, unveiling a selective vulnerability to metabolic and vascular factors of vertebro-basilar versus carotid territories [16].

Noteworthy, vascular-like lesions in AS patients seem to appear as early as 30 years. Better management of cardiac dysfunction will allow increasing survival of AS patients. The expected wider range of brain involvement will prove or deny the selective vulnerability of the vertebrobasilar territory. Recently, an ischemic stroke in a 21-year-old woman with Joubert syndrome has been reported, suggesting that this condition might be at higher risk for acute brain ischemia [17]. So far, no other ciliopathy has been associated with an increased vulnerability to vascular insults, but studies focused on this topic are lacking.

DTI evaluation added significant and unexpected information on white matter involvement in AS. Diffuse changes in radial diffusivity with almost no change in axial diffusivity were observed both in highly anisotropic regions, where the WM tracts are arranged as compact fiber bundles (e.g. the corpus callosum) and in the less anisotropic regions, where WM tracts are variably intermingled. Similar findings have been reported in other diseases characterized mostly by myelin derangement such as relapsing remitting multiple sclerosis and in animal models of dysmyelination [18]. White matter fractional anisotropy might be significantly and permanently altered by severe infantile myopathy-related hypoxia. However, neonatal hypoxia also alters permanently mean and axial diffusivity [19]. AS patients did not present with mean and axial diffusivity abnormalities (except from the fornix). Moreover, hypoxia usually presents with cerebellar white matter abnormalities [20] that were not found in AS patients.

According to these findings hypoxia does not seem to be able to explain supratentorial white matter changes in AS.

Recently, adolescent patients affected with obesity and metabolic syndrome have been shown to present small white matter clusters of decreased FA suggesting a pathogenic role of diabetes, hypertension and obesity in causing early structural brain changes [21]. These changes were limited (global volume of 4.47 mL), and not comparable with the widespread supratentorial involvement observed in our cohort.

Therefore, our findings uncover a diffuse and symmetric supratentorial myelination abnormality. Intriguingly, primary myelin issues have been already reported in the peripheral nervous system of AS patients. Delamination of the myelin sheaths was detected by transmission electron microscopy at the level of the gingiva [22]. Recently, it has been shown that the primary cilia of Schwann cells are the regulators of Hedgehog signaling-mediated myelination in the peripheral nervous system, [23,24] while genetic studies have shown that Sonic Hedgehog signaling at the level of primary cilia is essential for patterning the embryonic stem cells into oligodendrocytes [25,26]. Though the exact function of cilia in brain development is unclear, expression of genes involved in ciliary transport are seen in developing neural tissues. A recent MRI study investigated the brain in BBS, a ciliopathy that is clinically similar to AS with the exceptions of polydactyly and learning deficits [27]. By means of volumetric analisys, the study disclosed a significant decrease of white matter volume, suggesting a primary white matter involvement in this ciliopathy. Our findings on AS patients, another ciliopathy that does not feature impairment of cognitive performance, seem to confirm the pivotal role of cilia in the embryogenic mechanism leading to white matter formation.

So far, TBSS has not been applied to investigate white matter changes in BBS, Joubert Syndrome or Meckel-Gruber Syndrome; therefore, it is not possible to clarify whether the white matter involvement observed in AS were a feature shared by other ciliopathies.

The peculiar signal change of the fornix in AS patients is more difficult to unravel. In this region, AS patients had increased mean and radial diffusivity consistent with axonal involvement. It has been demonstrated that body mass index correlates positively with mean and radial diffusivity in the fornix [28]. Moreover, in several psychiatric diseases (e.g. schizophrenia, anorexia nervosa) an involvement of the fornix has been detected. Both these conditions (overweight or obesity and psychiatric disorders) have been observed in AS and might underlie fornix DTI abnormalities. In our group of AS patients ten of twelve were overweight or obese and four had psychiatric issues. On the other hand, the fornix involvement could represent the anatomical substrate leading to weight-related and psychiatry disorders that have already reported in this disease [2].

Finally, AS patients might take several drugs to control comorbidities. Some of these treatments could hypothetically have affected the abovementioned brain MRI findings.

Quetiapine has been shown to determine hippocampal volume loss in schizophrenic patients, [29] while risperidone has been reported to increase frontal lobe intracortical myelin volume as detected by means of inversion recovery and proton density weighted images [30]. In our study group only one patient was treated with the abovementioned drugs: the impact on the statistic should be, therefore, negligible if any.

Two patients received omega-3 fatty acids. Omega-3 fatty acids have been demonstrated to protect against cerobrovascular brain pathology and hippocampal volume loss [31,32]. Given that VBM data and conventional MRI disclosed mild hippocampal volume loss and increased white matter lesion burden in AS patients compared to age matched controls, a direct effect of omega-3 fatty acids in determining these findings is unlikely.

## Conclusions

Alström syndrome patients present with mild brain atrophy and vascular-like lesions at a very young age. Additionally, DTI reveals supratentorial dysmyelination which also entails the white matter that appears normal on conventional MRI sequences. The role of cilia in cerebral parenchymal changes and myelination needs further investigation.

## Competing interests

All the authors declare there are no conflict of interest.

## Authors’ contribution

RM and PM: study design, interpretation of data, revising the manuscript, study supervision VC: acquisition of data, interpretation of data and drafting the manuscript, BV: acquisition of data, drafting the manuscript, AF: analysis of data, interpretation of data, drafting the manuscript for content and revising the manuscript, NAG, MG and GJ: acquisition of data, drafting the manuscript, JDM and JKN: acquisition of data and revising the manuscript. All authors read and approved the final manuscript.

## Authors’ information

Renzo Manara, Pietro Maffei, Valentina Citton, Bettini Vera, Angela Favaro, Nella Augusta Greggio, Milan Gabriella, Gabrieli Joseph, Jan D Marshall and J K Naggert: These authors should be considered senior co-authors.
